# Byproduct identification: crystal structure of potassium 2-eth­oxy-2-oxo­acetate

**DOI:** 10.1107/S205698902600914X

**Published:** 2026-09-08

**Authors:** Shannon E. Creegan, James A. Ridenour, Robert J. Donnelly, Andrew T. Kerr

**Affiliations:** a US Naval Research Laboratory, Center for Biomolecular Science and Engineering, 4555 Overlook Ave., SW Washington, DC 20375, USA; b US Naval Research Laboratory Materials Chemistry and Dynamics Branch 4555 Overlook Ave SW Washington DC 20375 USA; c Department of Chemistry Washington College, 300 Washington Ave Chestertown MD 21620 USA; Vienna University of Technology, Austria

**Keywords:** crystal structure, byproduct identification, potassium oxalate-type salt, disorder

## Abstract

Potassium 2-eth­oxy-2-oxo­acetate, K^+^·C_4_H_5_O_4_^−^, was identified as a byproduct in the first step of synthesizing the shelf-stable salt potassium nitro­aceto­nitrile (KNAN). A single crystal of the title salt was obtained by recrystallization of the ethanol filtrate resulting from the purification of the intended product salt, potassium ethyl nitro­cyano­acetate. The asymmetric unit of K^+^·C_4_H_5_O_4_^−^, contains a single potassium cation and a 2-eth­oxy-2-oxo­acetate anion, the ethyl group of which is disordered over two sets of sites in a 0.792 (13):0.208 (13) ratio. The packing is dictated primarily by K—O electrostatic inter­actions and comprises eight bonded oxygen atoms per cation, with distances ranging from 2.706 (2) to 3.053 (4) Å.

## Abstract 2

The crystal structure of the title salt is made up from a potassium cation and a disordered 2-eth­oxy-2-oxo­acetate anion. The packing inter­actions are through electrostatic inter­actions between the potassium ions and the oxygen atoms.

## 1. Chemical context

**Figure 1:**
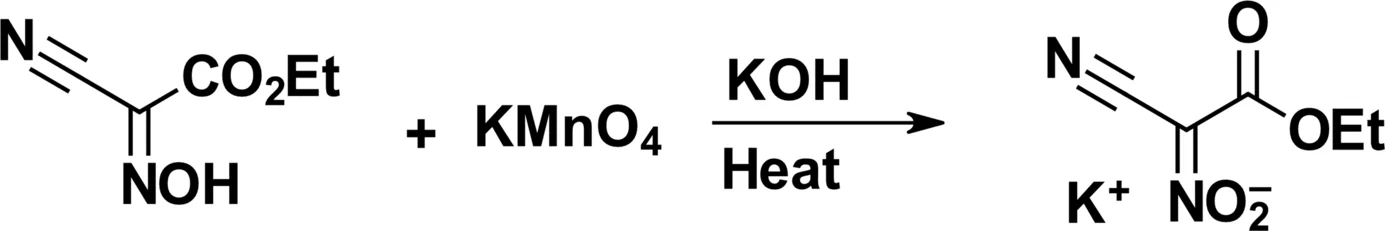
Synthesis scheme for the potassium salt of ethyl nitro­cyano­acetate.

Voinkov *et al.* (2016 ▸) reported an alternative synthesis method for the α-nitro­nitrile compound, nitro­aceto­nitrile, C_2_H_2_N_2_O_2_ (NAN). This synthesis was developed to bypass the greatest hazard of NAN synthesis, spontaneous explosion (Thomas, 2009 ▸), while improving the yield and purity of the NAN produced. The final product of the reaction detailed by Voinkov *et al.* (2016 ▸) is the potassium salt of nitro­aceto­nitrile (KNAN). Two key features of KNAN are its shelf-stability and that, as a salt, it is ready for immediate use in contrast to neutral NAN, which is unstable, requires careful storage, and needs to be deprotonated prior to use. The shelf-stable KNAN is produced in a two-step synthesis from ethyl (hy­droxy­imino)­cyano­acetate. During the first step of this process, ethyl (hy­droxy­imino)­cyano­acetate is oxidized with potassium permanganate in the presence of potassium hydroxide to form the salt potassium ethyl nitro­cyano­acetate in 63% yield (Fig. 1 ▸). It was in executing this first step that we identified the salt potassium 2-eth­oxy-2-oxo­acetate, the crystal structure of which is reported here.
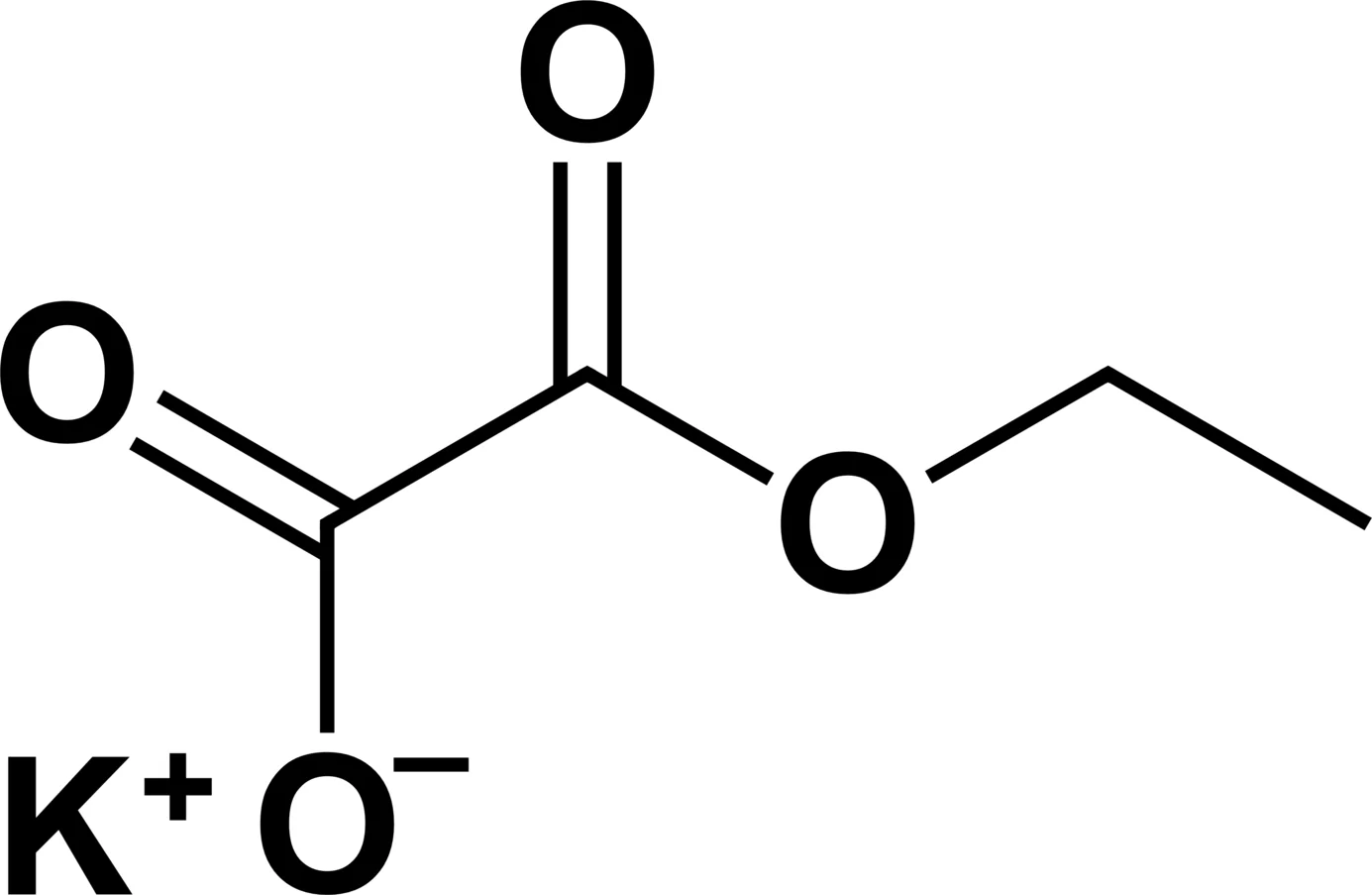


## 2. Structural commentary

**Figure 2:**
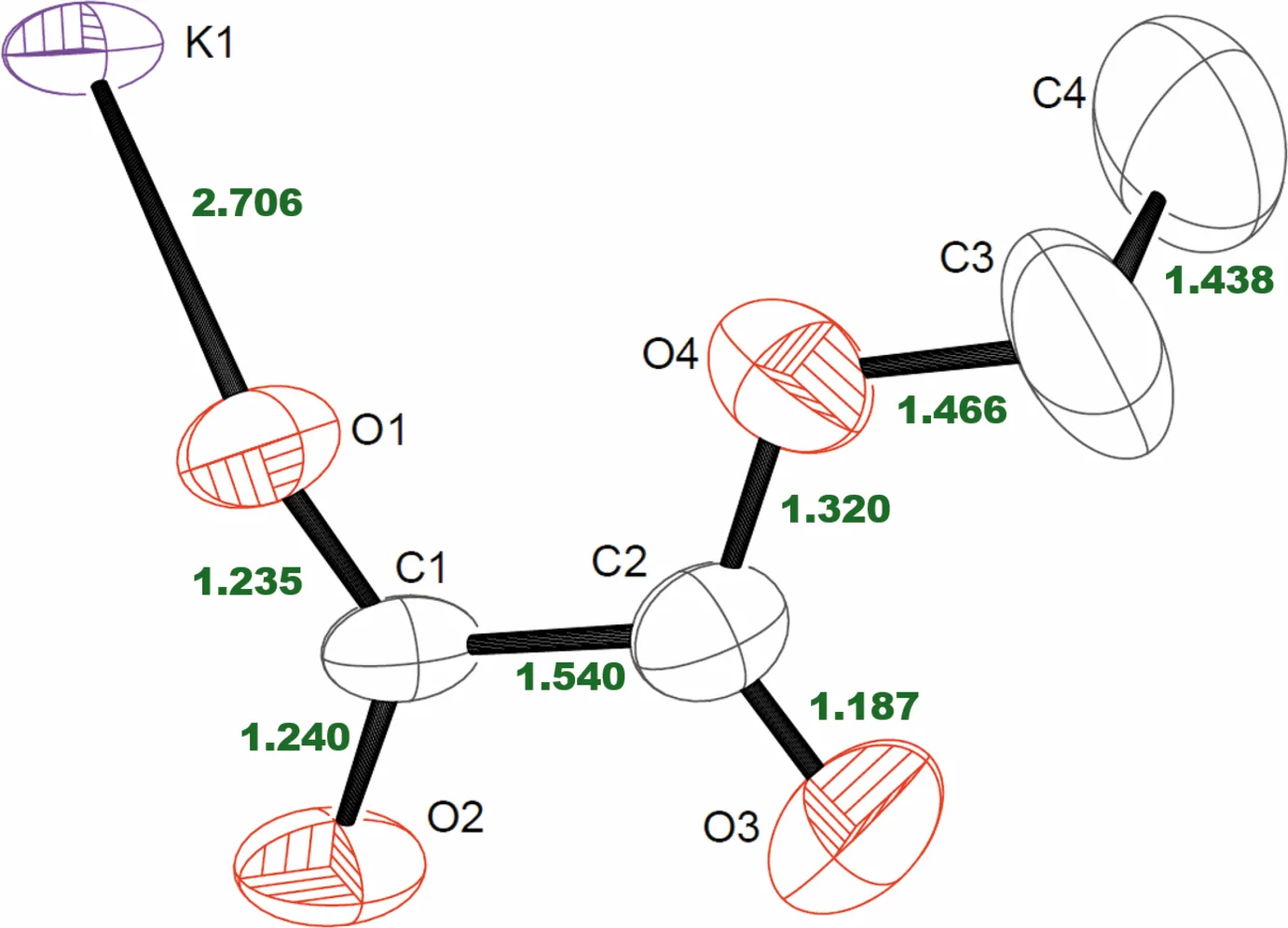
The asymmetric unit showing the potassium cation and the disordered 2-eth­oxy-2-oxo­acetate anion. Displacement ellipsoids are drawn at the 50% probability level; bonds lengths are detailed in green.

**Figure 3:**
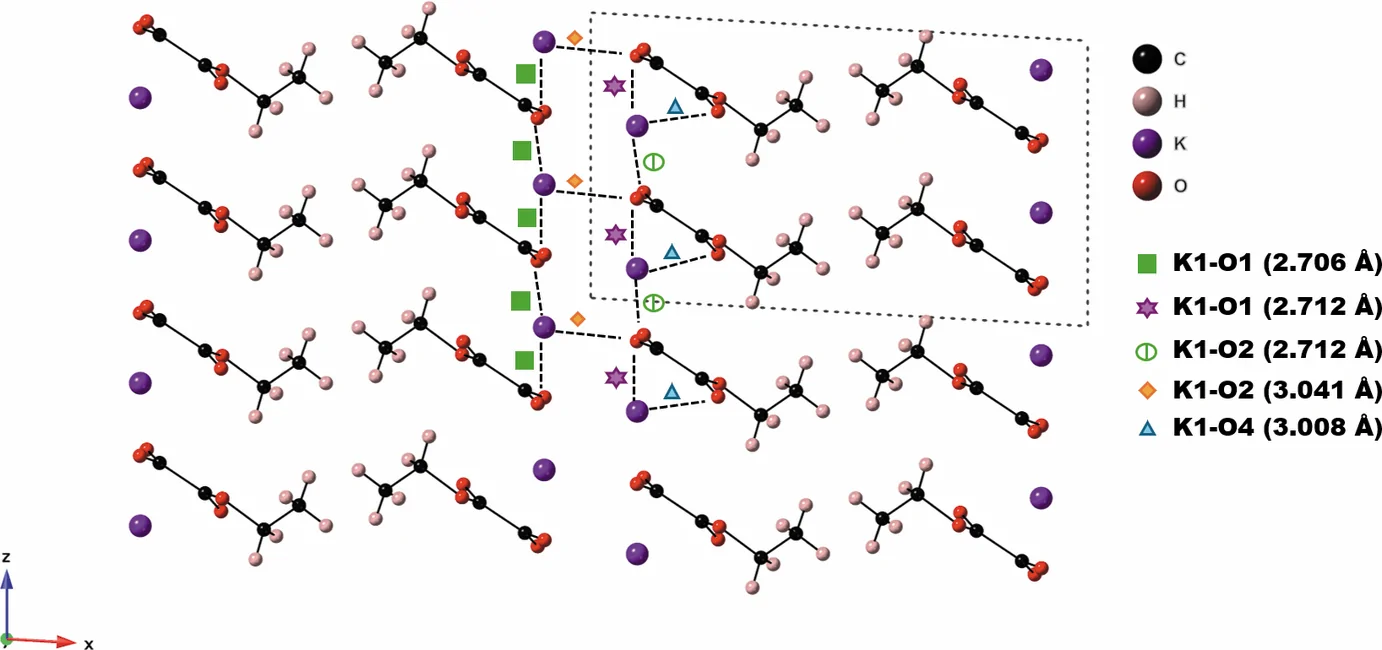
Ball-and-stick representation of the packing of 2-eth­oxy-2-oxo­acetate mol­ecules and potassium cations viewed down [010]. Several K—O distances are noted with black dotted lines.

The asymmetric unit of the title compound contains a single potassium cation and an anion, the ethyl fragment of which is disordered in a 0.792 (13):0.208 (13) ratio (Fig. 2 ▸). All bond lengths are in expected ranges when compared to similar potassium oxalate salt structures. The dihedral angle between the carb­oxy groups was determined to be 15.2 (6)°. The observed packing inter­actions (Fig. 3 ▸) of this polymeric salt structure are primarily electrostatic inter­actions between the potassium ion and the oxygen atoms of the 2-eth­oxy-2-oxo­acetate anion. These distances between potassium and oxygen range from 2.706 (2) to 3.053 (4) Å in length (see Table 1 ▸ for all K—O bonds).

**Figure 4:**
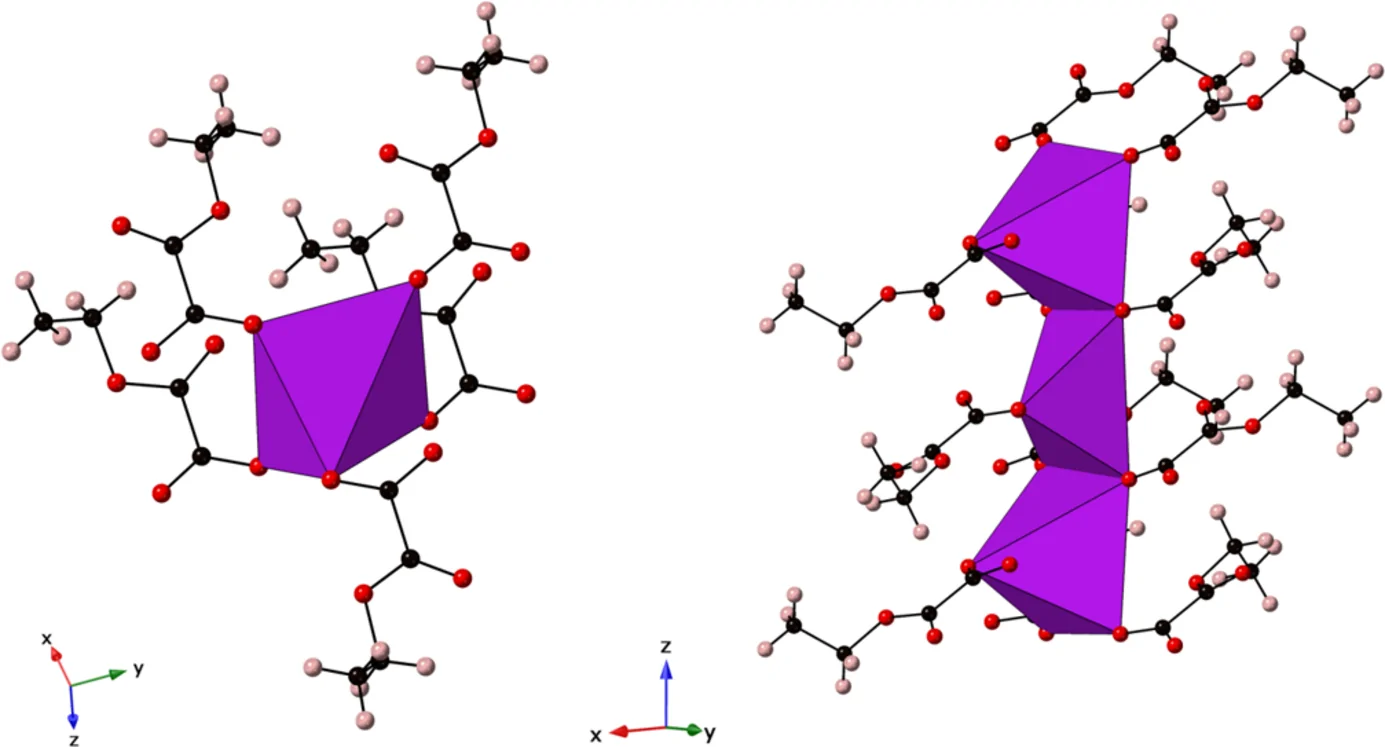
Ball-and-stick representation showing the distorted square-pyramidal coordination environment around the potassium cation as a purple polyhedron.

**Figure 6:**
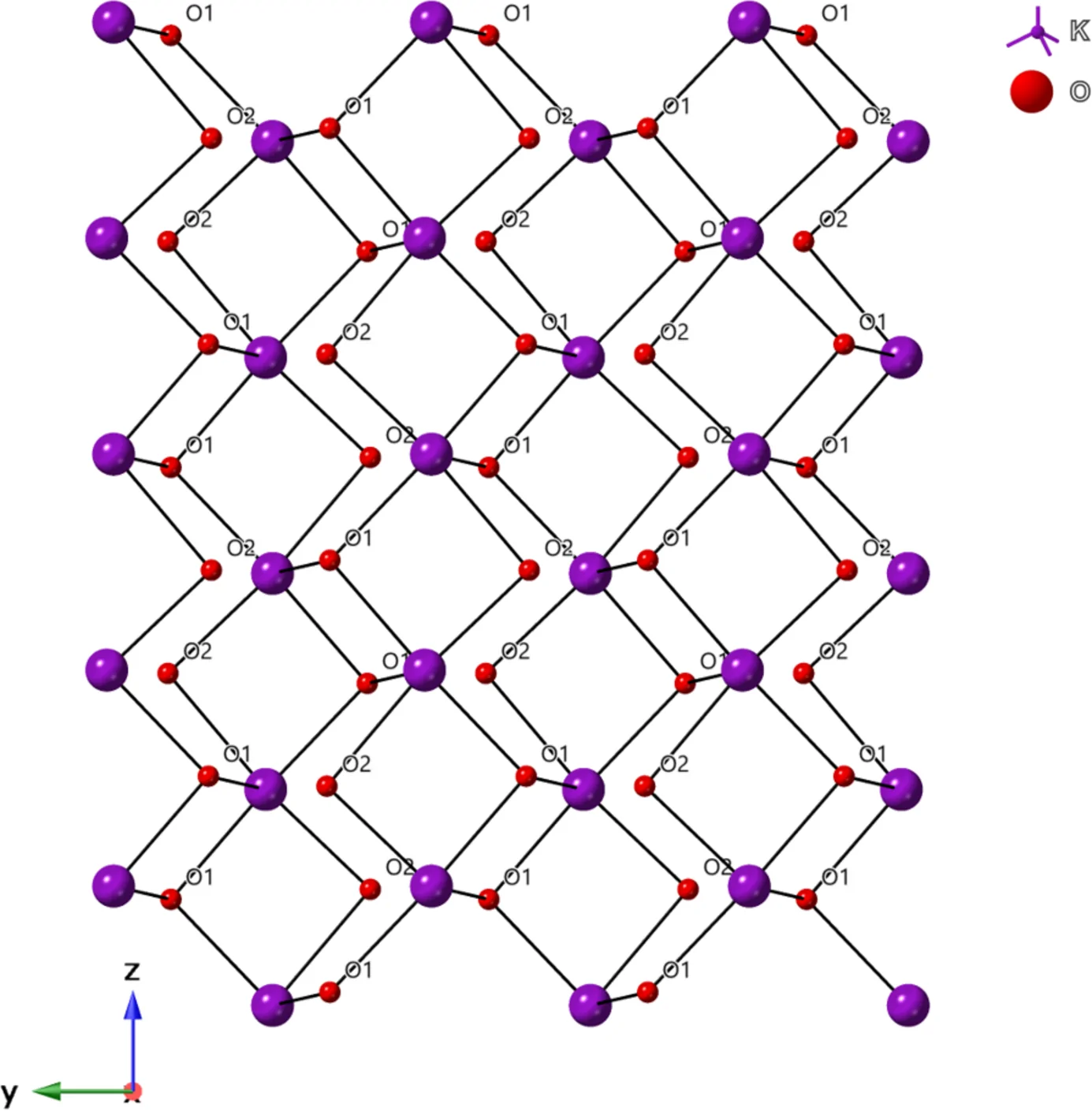
Inter­actions of O1 and O2 with potassium cations.

Taking into account K—O distances of less than 3.0 Å, the oxygen atoms arrange in form of a distorted square pyramid around K^+^, as represented by a purple polyhedron in Fig. 4 ▸. The extended structure reveals the formation of potassium-oxygen chains propagating parallel to [001] (Figs. 4 ▸, 5 ▸). Within a distance of 3.05 Å, atom O1 binds in a *μ*_3_-mode to three potassium ions while O2 inter­acts with two cations (Fig. 6 ▸). In contrast, both O3 and O4 only inter­act with one potassium ion each, and at longer distances (Table 1 ▸).

## 3. Database survey

**Figure 7:**
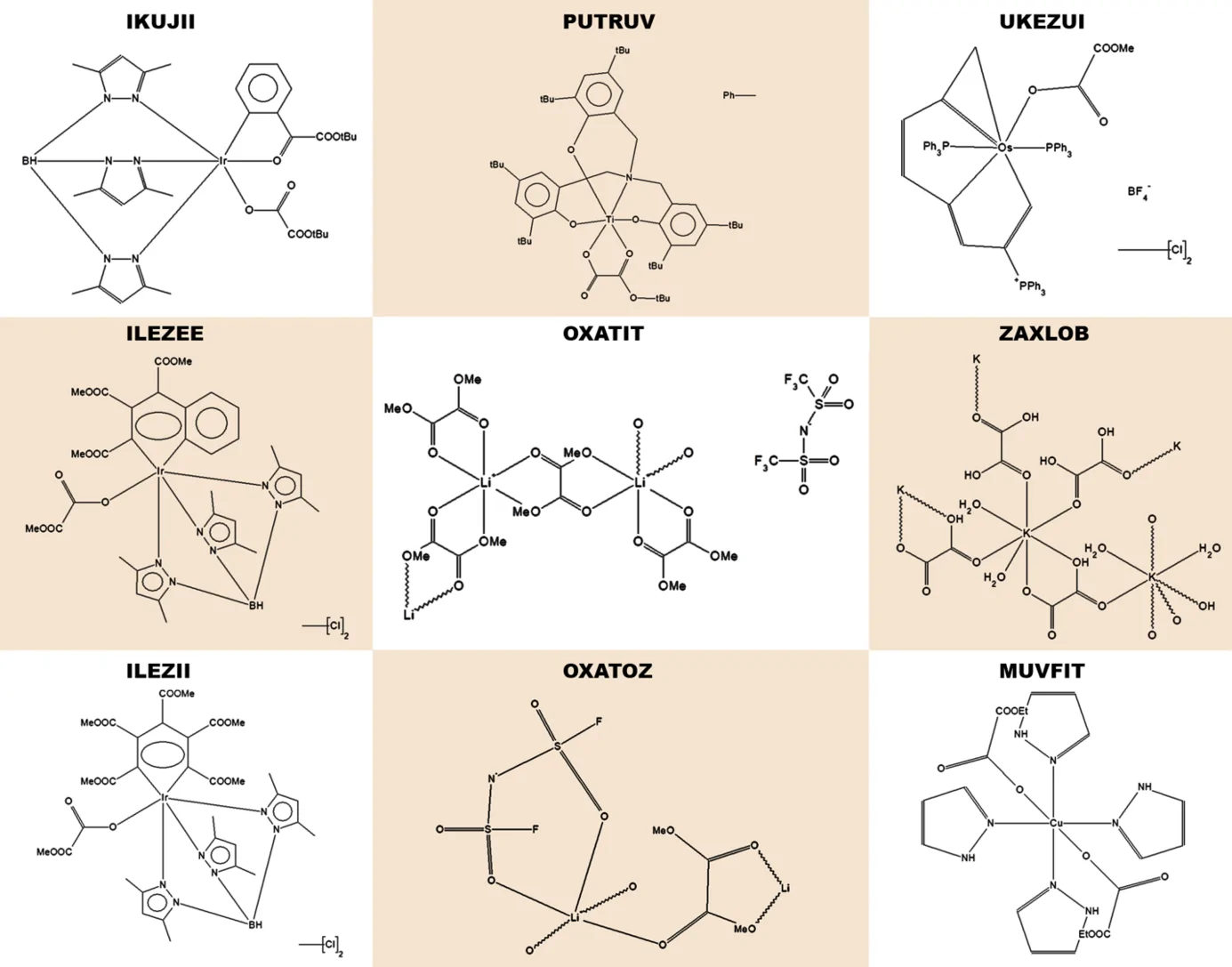
Chemical structures of the compounds referenced in the database survey.

A search of the Cambridge Structural Database (CSD, Version 5.46; Groom *et al.*, 2016 ▸) yielded entries containing either 2-eth­oxy-2-oxo­acetate bound to a metal or containing oxalate moieties bound to potassium as either a backbone structure or a metal coordinating ligand (Fig. 7 ▸). The most similar structures found were K_0.53_(NH_4_)_0.47_(H_2_C_2_O_4_)((HC_2_O_4_)H_2_O)_2_ (refcode ZAXLOB; Hamdouni *et al.*, 2011 ▸) and [Cu(EtOOC–COO)_2_(Hpz)_4_] (MUVFIT; Garau *et al.*, 2010 ▸). The ZAXLOB structure contains both oxalic acid and the hydrogenoxalate anion bound to potassium ions with K—O inter­action distances from 2.900 to 2.920 Å. MUVFIT contains two 2-eth­oxy-2-oxo­acetate units bound to copper(II) through a single oxygen per unit at 2.359 Å, a closer distance than any found in either the title structure or ZAXLOB structure to the potassium ion. An additional five CSD entries were found containing any metal bound to an oxalate group where a single oxygen atom is bonded to a methyl or *tert*-butyl group with another two entries having metal bound to oxalate where two oxygen atoms are bonded to methyl groups. The bound metals include titanium (PUTRUV, Okumura *et al.*, 2025 ▸), lithium (OXATIT and OXATOZ; Sun *et al.*, 2021 ▸), iridium (IKUJII, Padilla *et al.*, 2010 ▸; ILEZEE and ILEZII, Paneque *et al.*, 2003 ▸), and osmium (UKEZUI, Lin *et al.*, 2020 ▸).

## 4. Synthesis and crystallization

**Figure 8:**
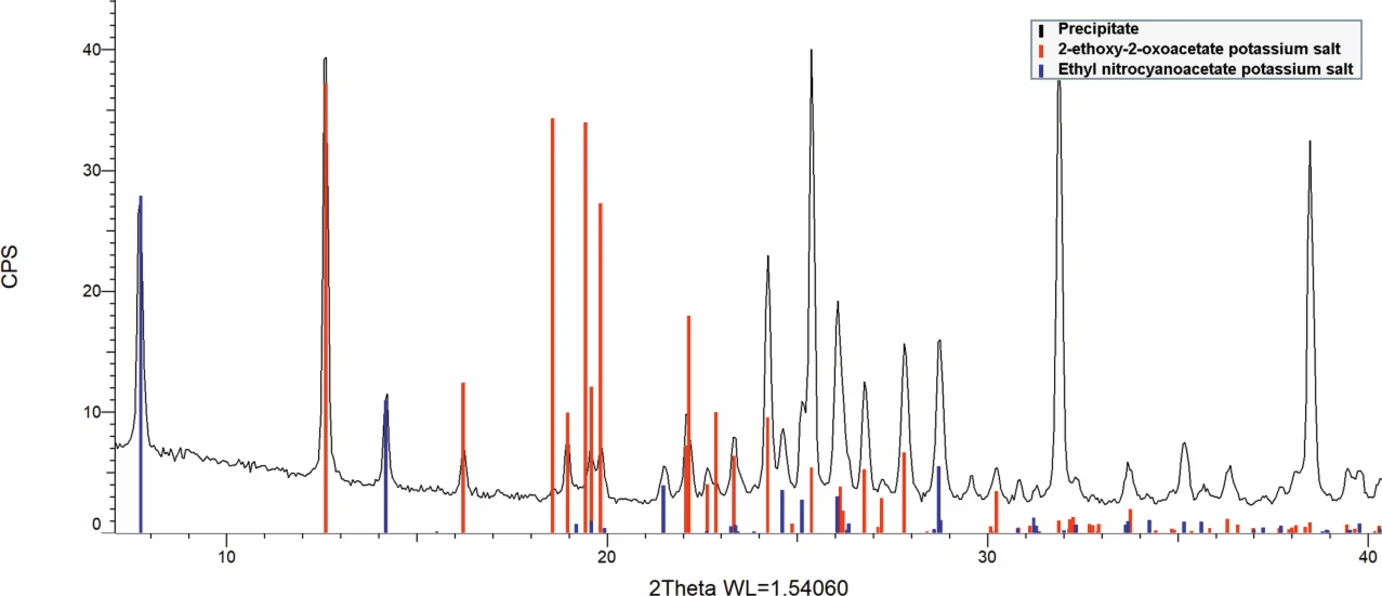
XRD pattern of isolated bulk material with comparison to the calculated pattern of the byproduct potassium 2-eth­oxy-2-oxo­acetate (red) and the intended product potassium ethyl nitro­cyano­acetate (blue). XRD pattern plotted with *DIFFRAC·EVA* with X-offset of 0.09° to correct for sample height.

A solution of KMnO_4_ (11.85 g, 75 mmol) in water (200 ml) was added dropwise over 1 h to a warm solution of KOH (0.9 g, 16 mmol) and ethyl (hy­droxy­imino)­cyano­acetate (7.15 g, 50.3 mmol) in water (140 ml). The solution was stirred overnight, until the KMnO_4_ was fully consumed as shown by the depletion of the dark purple coloring in favor of black. The solution was filtered, removing the black colored solids, and the filtrate concentrated on a rotary evaporator, producing pale-yellow crystalline material. The solids were washed with ethanol and isolated by filtration. The yellow filtrate was stored overnight in a refrigerator and additional material precipitated. From this precipitate, a crystal of the salt potassium 2-eth­oxy-2-oxo­acetate was isolated for single-crystal X-ray diffraction. Comparison of the calculated pattern to the experimental powder pattern of bulk material collected showed both the byproduct potassium 2-eth­oxy-2-oxo­acetate and potassium ethyl nitro­cyano­acetate present, next to some unassigned reflections (Fig. 8 ▸).

## 5. Refinement

Crystal data, data collection and structure refinement details are summarized in Table 2 ▸. A PART command in *SHELXL* (Sheldrick, 2015*b* ▸), accompanied by SIMU and RIGU restraints set to 0.01, were used to model the positional disorder within the ethyl group and resulted in a refined ratio of 0.792 (13):0.208 (2). Corresponding H atoms were included using a riding model for the methyl and methyl­ene groups.

## Supplementary Material

Crystal structure: contains datablock(s) I. DOI: 10.1107/S205698902600914X/wm5809sup1.cif

Structure factors: contains datablock(s) I. DOI: 10.1107/S205698902600914X/wm5809Isup2.hkl

Supporting information file. DOI: 10.1107/S205698902600914X/wm5809Isup3.cml

CCDC reference: 2584812

Additional supporting information:  crystallographic information; 3D view; checkCIF report

## Figures and Tables

**Figure 5:**
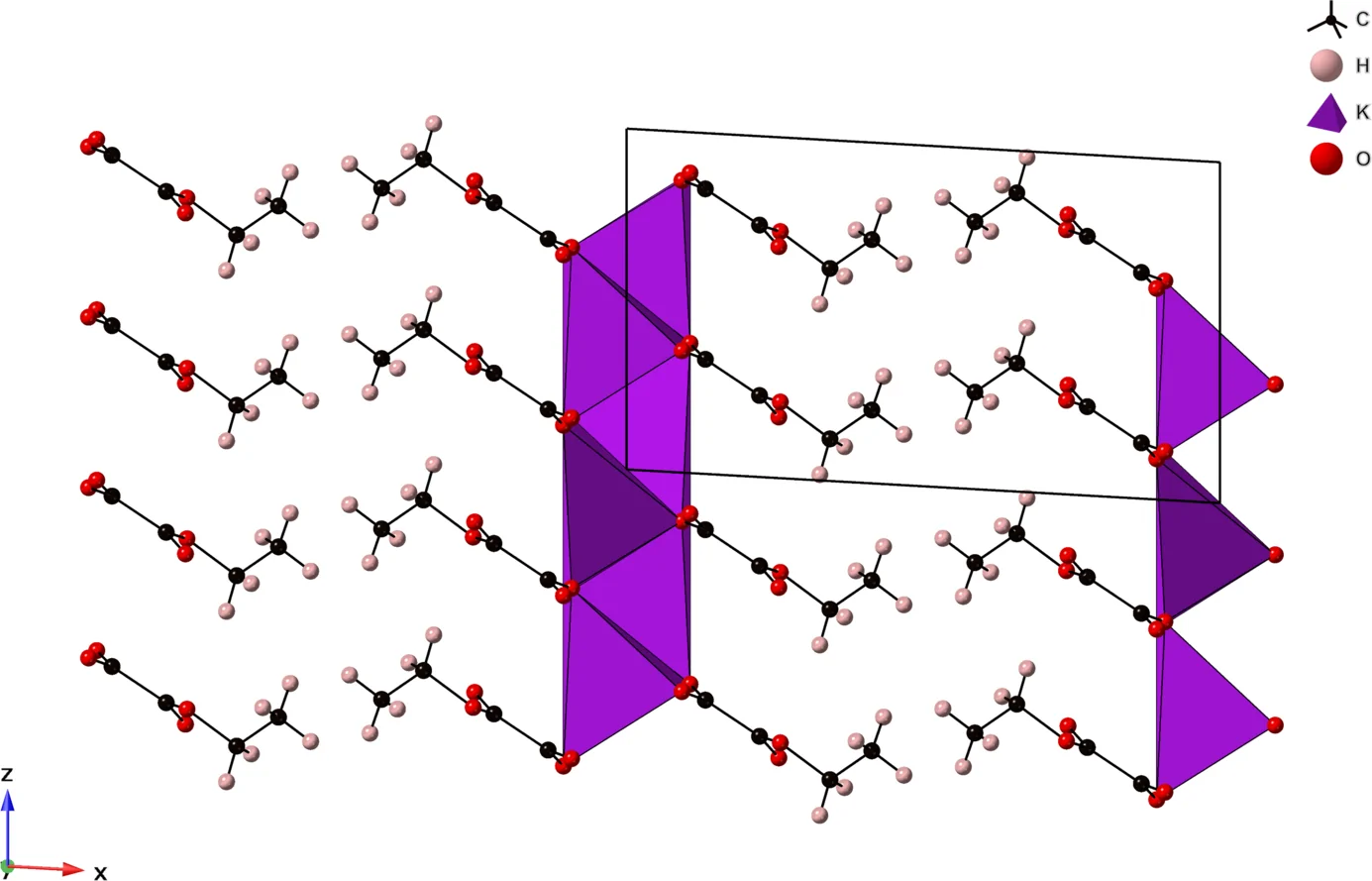
Ball and stick model of the extended structure of the title salt, showing the formation of potassium–oxygen chains propagating along [001] in polyhedral representation.

**Table 1:** Selected bond lengths (Å)

K1—O1	2.706 (2)	K1—O1^iv^	2.855 (3)
K1—O2^i^	2.712 (2)	K1—O4^ii^	3.008 (3)
K1—O1^ii^	2.741 (2)	K1—O2^iv^	3.041 (3)
K1—O2^iii^	2.828 (3)	K1—O3^iii^	3.053 (4)

Symmetry codes: (i) 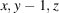; (ii) 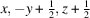; (iii) 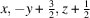; (iv) 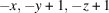.

**Table 2:** Experimental details

Crystal data	
Chemical formula	K^+^·C_4_H_5_O_4_^−^
*M*_r_	156.18
Crystal system, space group	Monoclinic, *P*2_1_/*c*
Temperature (K)	296
*a*, *b*, *c* (Å)	14.052 (2), 5.9280 (8), 8.0579 (10)
β (°)	93.242 (5)
*V* (Å^3^)	670.15 (16)
*Z*	4
Radiation type	Mo *K*α
μ (mm^−1^)	0.73
Crystal size (mm)	0.12 × 0.09 × 0.02

Data collection	
Diffractometer	Photon II CCD
Absorption correction	Multi-scan (*SADABS*; Krause *et al.*, 2015 ▸)
*T*_min_, *T*_max_	0.660, 0.746
No. of measured, independent and observed [*I* > 2σ(*I*)] reflections	10487, 1593, 1192
*R*_int_	0.059
(sin θ/λ)_max_ (Å^−1^)	0.657

Refinement	
*R*[*F*^2^ > 2σ(*F*^2^)], *wR*(*F*^2^), *S*	0.064, 0.150, 1.10
No. of reflections	1593
No. of parameters	103
No. of restraints	43
H-atom treatment	H-atom parameters constrained
Δρ_max_, Δρ_min_ (e Å^−3^)	0.42, −0.31

Computer programs: *APEX3* and *SAINT* (Bruker, 2019 ▸), *SHELXT* (Sheldrick, 2015*a* ▸), *SHELXL* (Sheldrick, 2015*b* ▸), *SHELXTL* (Sheldrick, 2008 ▸) and *publCIF* (Westrip, 2010 ▸).

## supplementary crystallographic information

### Potassium 2-ethoxy-2-oxoacetate Crystal data

**Table:**

K^+^·C_4_H_5_O_4_^−^	*F*(000) = 320
*M**_r_* = 156.18	*D*_x_ = 1.548 Mg m^−^^3^
Monoclinic, *P*2_1_/*c*	Mo *K*α radiation, λ = 0.71073 Å
*a* = 14.052 (2) Å	Cell parameters from 1931 reflections
*b* = 5.9280 (8) Å	θ = 2.9–27.2°
*c* = 8.0579 (10) Å	µ = 0.73 mm^−^^1^
β = 93.242 (5)°	*T* = 296 K
*V* = 670.15 (16) Å^3^	Plate, yellow
*Z* = 4	0.12 × 0.09 × 0.02 mm

### Potassium 2-ethoxy-2-oxoacetate Data collection

**Table:**

Photon II CCD diffractometer	1593 independent reflections
Radiation source: 1uS 3.0 microfocus	1192 reflections with *I* > 2σ(*I*)
Helios multilayer optics monochromator	*R*_int_ = 0.059
φ and ω scans	θ_max_ = 27.9°, θ_min_ = 2.9°
Absorption correction: multi-scan (*SADABS*; Krause *et al.*, 2015)	*h* = −17→18
*T*_min_ = 0.660, *T*_max_ = 0.746	*k* = −7→7
10487 measured reflections	*l* = −10→10

### Potassium 2-ethoxy-2-oxoacetate Refinement

**Table:**

Refinement on *F*^2^	Primary atom site location: intrinsic phasing
Least-squares matrix: full	Hydrogen site location: inferred from neighbouring sites
*R*[*F*^2^ > 2σ(*F*^2^)] = 0.064	H-atom parameters constrained
*wR*(*F*^2^) = 0.150	*w* = 1/[σ^2^(*F*_o_^2^) + (0.0533*P*)^2^ + 0.7606*P*] where *P* = (*F*_o_^2^ + 2*F*_c_^2^)/3
*S* = 1.10	(Δ/σ)_max_ = 0.003
1593 reflections	Δρ_max_ = 0.42 e Å^−^^3^
103 parameters	Δρ_min_ = −0.31 e Å^−^^3^
43 restraints	

### Potassium 2-ethoxy-2-oxoacetate Special details

**Table:**

Geometry. All esds (except the esd in the dihedral angle between two l.s. planes) are estimated using the full covariance matrix. The cell esds are taken into account individually in the estimation of esds in distances, angles and torsion angles; correlations between esds in cell parameters are only used when they are defined by crystal symmetry. An approximate (isotropic) treatment of cell esds is used for estimating esds involving l.s. planes.

### Potassium 2-ethoxy-2-oxoacetate Fractional atomic coordinates and isotropic or equivalent isotropic displacement parameters (Å^2^)

**Table:**

	*x*	*y*	*z*	*U*_iso_*/*U*_eq_	Occ. (<1)
K1	0.09360 (6)	0.26080 (10)	0.61193 (8)	0.0455 (3)	
O1	0.0930 (2)	0.5589 (4)	0.3575 (3)	0.0552 (7)	
O2	0.1067 (2)	0.9317 (4)	0.3806 (3)	0.0650 (8)	
O3	0.2563 (3)	0.9045 (6)	0.1792 (5)	0.0985 (12)	
O4	0.2588 (2)	0.5356 (5)	0.2237 (4)	0.0761 (9)	
C1	0.1325 (3)	0.7420 (5)	0.3364 (4)	0.0436 (8)	
C2	0.2238 (3)	0.7407 (6)	0.2392 (5)	0.0552 (9)	
C3	0.3425 (7)	0.521 (2)	0.1236 (14)	0.114 (3)	0.792 (13)
H3A	0.325405	0.450607	0.017359	0.137*	0.792 (13)
H3B	0.367178	0.670628	0.103228	0.137*	0.792 (13)
C4	0.4131 (6)	0.388 (2)	0.2152 (16)	0.144 (4)	0.792 (13)
H4A	0.467205	0.366411	0.149261	0.216*	0.792 (13)
H4B	0.386482	0.243678	0.241020	0.216*	0.792 (13)
H4C	0.432783	0.464288	0.316375	0.216*	0.792 (13)
C3A	0.376 (3)	0.547 (8)	0.198 (6)	0.122 (5)	0.208 (13)
H3AA	0.395966	0.686855	0.149019	0.147*	0.208 (13)
H3AB	0.414355	0.513027	0.298734	0.147*	0.208 (13)
C4A	0.369 (3)	0.362 (7)	0.083 (5)	0.125 (6)	0.208 (13)
H4AA	0.345767	0.416681	−0.024271	0.187*	0.208 (13)
H4AB	0.325420	0.251309	0.121351	0.187*	0.208 (13)
H4AC	0.430521	0.295372	0.074146	0.187*	0.208 (13)

### Potassium 2-ethoxy-2-oxoacetate Atomic displacement parameters (Å^2^)

**Table:**

	*U*^11^	*U*^22^	*U*^33^	*U*^12^	*U*^13^	*U*^23^
K1	0.0772 (6)	0.0234 (3)	0.0364 (4)	0.0041 (3)	0.0082 (3)	0.0011 (3)
O1	0.0809 (19)	0.0348 (12)	0.0516 (14)	−0.0030 (12)	0.0190 (13)	0.0021 (10)
O2	0.103 (2)	0.0317 (13)	0.0605 (17)	0.0068 (13)	0.0097 (16)	−0.0088 (11)
O3	0.103 (3)	0.068 (2)	0.128 (3)	−0.0207 (19)	0.040 (2)	0.016 (2)
O4	0.078 (2)	0.0634 (18)	0.089 (2)	0.0200 (15)	0.0244 (17)	0.0030 (16)
C1	0.069 (2)	0.0284 (14)	0.0332 (14)	0.0018 (15)	0.0006 (14)	−0.0003 (12)
C2	0.066 (2)	0.0461 (19)	0.053 (2)	−0.0061 (18)	0.0035 (18)	−0.0002 (16)
C3	0.074 (6)	0.145 (7)	0.125 (8)	0.045 (5)	0.029 (5)	0.008 (6)
C4	0.082 (6)	0.163 (8)	0.188 (10)	0.035 (6)	0.021 (6)	0.045 (8)
C3A	0.080 (10)	0.147 (10)	0.144 (12)	0.047 (9)	0.035 (9)	0.015 (10)
C4A	0.081 (12)	0.151 (12)	0.146 (13)	0.033 (11)	0.032 (11)	0.018 (12)

### Potassium 2-ethoxy-2-oxoacetate Geometric parameters (Å, º)

**Table:**

K1—O1	2.706 (2)	O4—C3	1.465 (9)
K1—O2^i^	2.712 (2)	O4—C3A	1.67 (4)
K1—O1^ii^	2.741 (2)	C1—C2	1.540 (5)
K1—O2^iii^	2.828 (3)	C3—C4	1.439 (12)
K1—O1^iv^	2.855 (3)	C3—H3A	0.9700
K1—O4^ii^	3.008 (3)	C3—H3B	0.9700
K1—O2^iv^	3.041 (3)	C4—H4A	0.9600
K1—O3^iii^	3.053 (4)	C4—H4B	0.9600
K1—C1^iv^	3.228 (4)	C4—H4C	0.9600
K1—C1^iii^	3.485 (3)	C3A—C4A	1.44 (2)
K1—C1^ii^	3.513 (3)	C3A—H3AA	0.9700
K1—K1^ii^	4.0310 (5)	C3A—H3AB	0.9700
O1—C1	1.235 (4)	C4A—H4AA	0.9600
O2—C1	1.240 (4)	C4A—H4AB	0.9600
O3—C2	1.187 (5)	C4A—H4AC	0.9600
O4—C2	1.320 (4)		
			
O1—K1—O2^i^	86.93 (8)	C1^iv^—K1—K1^ii^	79.35 (6)
O1—K1—O1^ii^	176.99 (7)	C1^iii^—K1—K1^ii^	61.12 (5)
O2^i^—K1—O1^ii^	90.11 (8)	C1^ii^—K1—K1^ii^	57.81 (5)
O1—K1—O2^iii^	98.99 (7)	C1—O1—K1	134.1 (2)
O2^i^—K1—O2^iii^	170.36 (12)	C1—O1—K1^v^	119.2 (2)
O1^ii^—K1—O2^iii^	84.01 (7)	K1—O1—K1^v^	95.47 (7)
O1—K1—O1^iv^	81.80 (8)	C1—O1—K1^iv^	96.1 (2)
O2^i^—K1—O1^iv^	115.28 (9)	K1—O1—K1^iv^	98.20 (8)
O1^ii^—K1—O1^iv^	98.99 (6)	K1^v^—O1—K1^iv^	111.19 (10)
O2^iii^—K1—O1^iv^	73.33 (8)	C1—O2—K1^vi^	151.9 (2)
O1—K1—O4^ii^	125.26 (8)	C1—O2—K1^vii^	111.8 (2)
O2^i^—K1—O4^ii^	72.80 (9)	K1^vi^—O2—K1^vii^	93.36 (7)
O1^ii^—K1—O4^ii^	54.12 (8)	C1—O2—K1^iv^	87.2 (2)
O2^iii^—K1—O4^ii^	97.56 (9)	K1^vi^—O2—K1^iv^	99.06 (9)
O1^iv^—K1—O4^ii^	152.84 (8)	K1^vii^—O2—K1^iv^	103.70 (9)
O1—K1—O2^iv^	107.27 (8)	C2—O3—K1^vii^	107.6 (3)
O2^i^—K1—O2^iv^	80.94 (9)	C2—O4—C3	114.9 (6)
O1^ii^—K1—O2^iv^	71.67 (8)	C2—O4—C3A	110.5 (16)
O2^iii^—K1—O2^iv^	104.30 (6)	C2—O4—K1^v^	106.3 (3)
O1^iv^—K1—O2^iv^	44.20 (7)	C3—O4—K1^v^	115.5 (6)
O4^ii^—K1—O2^iv^	118.32 (8)	O1—C1—O2	128.0 (4)
O1—K1—O3^iii^	71.36 (10)	O1—C1—C2	117.4 (3)
O2^i^—K1—O3^iii^	120.69 (9)	O2—C1—C2	114.6 (3)
O1^ii^—K1—O3^iii^	110.74 (10)	O1—C1—K1^iv^	61.6 (2)
O2^iii^—K1—O3^iii^	55.21 (9)	O2—C1—K1^iv^	70.2 (2)
O1^iv^—K1—O3^iii^	115.07 (8)	C2—C1—K1^iv^	156.9 (2)
O4^ii^—K1—O3^iii^	76.63 (9)	O1—C1—K1^vii^	139.1 (3)
O2^iv^—K1—O3^iii^	157.70 (8)	O2—C1—K1^vii^	48.87 (17)
O1—K1—C1^iv^	98.06 (8)	C2—C1—K1^vii^	81.45 (18)
O2^i^—K1—C1^iv^	100.99 (9)	K1^iv^—C1—K1^vii^	86.81 (8)
O1^ii^—K1—C1^iv^	81.96 (8)	O1—C1—K1^v^	42.90 (15)
O2^iii^—K1—C1^iv^	85.78 (8)	O2—C1—K1^v^	151.4 (3)
O1^iv^—K1—C1^iv^	22.36 (7)	C2—C1—K1^v^	81.02 (18)
O4^ii^—K1—C1^iv^	134.99 (8)	K1^iv^—C1—K1^v^	86.35 (8)
O2^iv^—K1—C1^iv^	22.57 (6)	K1^vii^—C1—K1^v^	115.78 (9)
O3^iii^—K1—C1^iv^	135.50 (8)	O3—C2—O4	124.1 (4)
O1—K1—C1^iii^	80.47 (7)	O3—C2—C1	123.6 (4)
O2^i^—K1—C1^iii^	161.88 (9)	O4—C2—C1	112.2 (3)
O1^ii^—K1—C1^iii^	102.53 (7)	C4—C3—O4	107.6 (8)
O2^iii^—K1—C1^iii^	19.29 (7)	C4—C3—H3A	110.2
O1^iv^—K1—C1^iii^	75.92 (8)	O4—C3—H3A	110.2
O4^ii^—K1—C1^iii^	104.05 (9)	C4—C3—H3B	110.2
O2^iv^—K1—C1^iii^	115.18 (8)	O4—C3—H3B	110.2
O3^iii^—K1—C1^iii^	42.61 (9)	H3A—C3—H3B	108.5
C1^iv^—K1—C1^iii^	93.68 (9)	C3—C4—H4A	109.5
O1—K1—C1^ii^	159.67 (7)	C3—C4—H4B	109.5
O2^i^—K1—C1^ii^	74.27 (8)	H4A—C4—H4B	109.5
O1^ii^—K1—C1^ii^	17.87 (7)	C3—C4—H4C	109.5
O2^iii^—K1—C1^ii^	98.69 (7)	H4A—C4—H4C	109.5
O1^iv^—K1—C1^ii^	112.97 (8)	H4B—C4—H4C	109.5
O4^ii^—K1—C1^ii^	41.87 (8)	C4A—C3A—O4	91 (3)
O2^iv^—K1—C1^ii^	77.81 (8)	C4A—C3A—H3AA	113.5
O3^iii^—K1—C1^ii^	111.51 (10)	O4—C3A—H3AA	113.5
C1^iv^—K1—C1^ii^	93.16 (8)	C4A—C3A—H3AB	113.5
C1^iii^—K1—C1^ii^	115.79 (9)	O4—C3A—H3AB	113.5
O1—K1—K1^ii^	141.05 (6)	H3AA—C3A—H3AB	110.8
O2^i^—K1—K1^ii^	131.92 (6)	C3A—C4A—H4AA	109.5
O1^ii^—K1—K1^ii^	41.94 (5)	C3A—C4A—H4AB	109.5
O2^iii^—K1—K1^ii^	42.18 (5)	H4AA—C4A—H4AB	109.5
O1^iv^—K1—K1^ii^	82.75 (5)	C3A—C4A—H4AC	109.5
O4^ii^—K1—K1^ii^	74.10 (6)	H4AA—C4A—H4AC	109.5
O2^iv^—K1—K1^ii^	85.18 (5)	H4AB—C4A—H4AC	109.5
O3^iii^—K1—K1^ii^	83.42 (8)		
			
K1—O1—C1—O2	−83.4 (5)	K1^vii^—O2—C1—K1^v^	−66.8 (5)
K1^v^—O1—C1—O2	142.7 (3)	K1^iv^—O2—C1—K1^v^	36.9 (5)
K1^iv^—O1—C1—O2	24.2 (4)	K1^vii^—O3—C2—O4	151.2 (4)
K1—O1—C1—C2	98.6 (3)	K1^vii^—O3—C2—C1	−25.4 (5)
K1^v^—O1—C1—C2	−35.4 (4)	C3—O4—C2—O3	−0.5 (8)
K1^iv^—O1—C1—C2	−153.8 (3)	C3A—O4—C2—O3	28.4 (19)
K1—O1—C1—K1^iv^	−107.6 (3)	K1^v^—O4—C2—O3	−129.6 (4)
K1^v^—O1—C1—K1^iv^	118.4 (2)	C3—O4—C2—C1	176.4 (6)
K1—O1—C1—K1^vii^	−151.46 (18)	C3A—O4—C2—C1	−154.7 (18)
K1^v^—O1—C1—K1^vii^	74.6 (4)	K1^v^—O4—C2—C1	47.4 (3)
K1^iv^—O1—C1—K1^vii^	−43.8 (3)	O1—C1—C2—O3	162.8 (4)
K1—O1—C1—K1^v^	134.0 (4)	O2—C1—C2—O3	−15.6 (6)
K1^iv^—O1—C1—K1^v^	−118.4 (2)	K1^iv^—C1—C2—O3	81.5 (7)
K1^vi^—O2—C1—O1	81.6 (7)	K1^vii^—C1—C2—O3	21.2 (4)
K1^vii^—O2—C1—O1	−126.2 (3)	K1^v^—C1—C2—O3	139.2 (4)
K1^iv^—O2—C1—O1	−22.6 (4)	O1—C1—C2—O4	−14.2 (5)
K1^vi^—O2—C1—C2	−100.3 (5)	O2—C1—C2—O4	167.5 (3)
K1^vii^—O2—C1—C2	51.9 (4)	K1^iv^—C1—C2—O4	−95.4 (6)
K1^iv^—O2—C1—C2	155.5 (3)	K1^vii^—C1—C2—O4	−155.7 (3)
K1^vi^—O2—C1—K1^iv^	104.1 (5)	K1^v^—C1—C2—O4	−37.7 (3)
K1^vii^—O2—C1—K1^iv^	−103.65 (16)	C2—O4—C3—C4	132.1 (10)
K1^vi^—O2—C1—K1^vii^	−152.2 (6)	K1^v^—O4—C3—C4	−103.5 (11)
K1^iv^—O2—C1—K1^vii^	103.65 (16)	C2—O4—C3A—C4A	−143 (3)
K1^vi^—O2—C1—K1^v^	141.0 (4)	K1^v^—O4—C3A—C4A	5 (5)

Symmetry codes: (i) *x*, *y*−1, *z*; (ii) *x*, −*y*+1/2, *z*+1/2; (iii) *x*, −*y*+3/2, *z*+1/2; (iv) −*x*, −*y*+1, −*z*+1; (v) *x*, −*y*+1/2, *z*−1/2; (vi) *x*, *y*+1, *z*; (vii) *x*, −*y*+3/2, *z*−1/2.

## References

[bb1] Bruker (2019). *APEX3* and *SAINT*. Bruker AXS Inc., Madison, Wisconsin, USA.

[bb2] Garau, F., Monari, M., Pandolfo, L., Pettinari, C. & Venzo, A. (2010). *CrystEngComm* **12**, 1217–1226.

[bb3] Groom, C. R., Bruno, I. J., Lightfoot, M. P. & Ward, S. C. (2016). *Acta Cryst.* B**72**, 171–179. 10.1107/S2052520616003954 PMC4822653 27048719

[bb4] Hamdouni, M., Agengui, L., Walha, S., Kabadou, A. & Ben Salah, A. (2011). *J. Chem. Crystallogr.* **41**, 1742–1750.

[bb5] Krause, L., Herbst-Irmer, R., Sheldrick, G. M. & Stalke, D. (2015). *J. Appl. Cryst.* **48**, 3–10. 10.1107/S1600576714022985 PMC4453166 26089746

[bb6] Lin, J., Xu, Q., Lin, X., Hua, Y., Chen, D., Ruan, Y., Zhang, H. & Xia, H. (2020). *Chin. J. Chem.* **38**, 1273–1279.

[bb7] Okumura, A., Spaniol, T. P. & Okuda, J. (2025). *Z. Anorg. Allg. Chem.* **651**, e202400209.

[bb8] Padilla, R., Salazar, V., Paneque, M., Alvarado-Rodríguez, J. G., Tamariz, J., Pacheco-Cuvas, H. & Vattier, F. (2010). *Organometallics* **29**, 2835–2838.

[bb9] Paneque, M., Posadas, C. M., Poveda, M. L., Rendón, N., Salazar, V., Oñate, E. & Mereiter, K. (2003). *J. Am. Chem. Soc.* **125**, 9898–9899. 10.1021/ja035655p 12914433

[bb10] Sheldrick, G. M. (2008). *Acta Cryst.* A**64**, 112–122. 10.1107/S0108767307043930 18156677

[bb11] Sheldrick, G. M. (2015*a*). *Acta Cryst.* A**71**, 3–8.

[bb12] Sheldrick, G. M. (2015*b*). *Acta Cryst.* C**71**, 3–8.

[bb13] Sun, H., Xie, X., Huang, Q., Wang, Z., Chen, K., Li, X., Gao, J., Li, Y., Li, H., Qiu, J. & Zhou, W. (2021). *Angew. Chem. Int. Ed.* **60**, 18335–18343. 10.1002/anie.202107667 34157197

[bb14] Thomas, C. J. (2009). *Chem. Eng. News* **87**, 4.

[bb15] Voinkov, E. K., Ulomskiy, E. N., Rusinov, V. L., Savateev, K. V., Fedotov, V. V., Gorbunov, E. B., Isenov, M. & Eltsov, O. S. (2016). *Mendeleev Commun.* **26**, 172–173.

[bb16] Westrip, S. P. (2010). *J. Appl. Cryst.* **43**, 920–925.

